# Retrofit Weight-Loss Outcomes at 6, 12, and 24 Months and Characteristics of 12-Month High Performers: A Retrospective Analysis

**DOI:** 10.2196/mhealth.5873

**Published:** 2016-08-22

**Authors:** Stefanie Painter, Gary Ditsch, Rezwan Ahmed, Nicholas Buck Hanson, Kevin Kachin, Jan Berger

**Affiliations:** ^1^ Retrofit, Inc Chicago, IL United States; ^2^ Health Intelligence Partners CEO Chicago, IL United States

**Keywords:** behavior, body mass index, BMI, engagement, fitness, self-monitoring, obesity, overweight, weight loss

## Abstract

**Background:**

Obesity is the leading cause of preventable death costing the health care system billions of dollars. Combining self-monitoring technology with personalized behavior change strategies results in clinically significant weight loss. However, there is a lack of real-world outcomes in commercial weight-loss program research.

**Objective:**

Retrofit is a personalized weight management and disease-prevention solution. This study aimed to report Retrofit’s weight-loss outcomes at 6, 12, and 24 months and characterize behaviors, age, and sex of high-performing participants who achieved weight loss of 10% or greater at 12 months.

**Methods:**

A retrospective analysis was performed from 2011 to 2014 using 2720 participants enrolled in a Retrofit weight-loss program. Participants had a starting body mass index (BMI) of >25 kg/m² and were at least 18 years of age. Weight measurements were assessed at 6, 12, and 24 months in the program to evaluate change in body weight, BMI, and percentage of participants who achieved 5% or greater weight loss. A secondary analysis characterized high-performing participants who lost ≥10% of their starting weight (n=238). Characterized behaviors were evaluated, including self-monitoring through weigh-ins, number of days wearing an activity tracker, daily step count average, and engagement through coaching conversations via Web-based messages, and number of coaching sessions attended.

**Results:**

Average weight loss at 6 months was −5.55% for male and −4.86% for female participants. Male and female participants had an average weight loss of −6.28% and −5.37% at 12 months, respectively. Average weight loss at 24 months was −5.03% and −3.15% for males and females, respectively. Behaviors of high-performing participants were assessed at 12 months. Number of weigh-ins were greater in high-performing male (197.3 times vs 165.4 times, *P*=.001) and female participants (222 times vs 167 times, *P*<.001) compared with remaining participants. Total activity tracker days and average steps per day were greater in high-performing females (304.7 vs 266.6 days, *P*<.001; 8380.9 vs 7059.7 steps, *P*<.001, respectively) and males (297.1 vs 255.3 days, *P*<.001; 9099.3 vs 8251.4 steps, *P*=.008, respectively). High-performing female participants had significantly more coaching conversations via Web-based messages than remaining female participants (341.4 vs 301.1, *P*=.03), as well as more days with at least one such electronic message (118 vs 108 days, *P*=.03). High-performing male participants displayed similar behavior.

**Conclusions:**

Participants on the Retrofit program lost an average of −5.21% at 6 months, −5.83% at 12 months, and −4.09% at 24 months. High-performing participants show greater adherence to self-monitoring behaviors of weighing in, number of days wearing an activity tracker, and average number of steps per day. Female high performers have higher coaching engagement through conversation days and total number of coaching conversations.

## Introduction

Obesity is the leading cause of preventable death in the world, yet it continues to remain a crisis in the United States with two-thirds of the American adult population overweight or obese [1]. The overweight population in the United States has nearly doubled, while obesity rates have nearly tripled in the past 50 years [2]. Direct and indirect health care costs for preventable chronic disease, including obesity-related diseases such as heart disease and diabetes, range from US $147 billion to US $215 billion per year [3,4]. On average, women report trying to lose weight 7 times in their lifetime, whereas men report an average of 3.6 times [5]. In fact, Americans spent US $2.5 billion on weight-loss programs and products in 2014 [6].

The Affordable Care Act encourages employee wellness programs designed to increase health knowledge and skills to promote healthy behaviors, which can aid in the reduction of health care costs incurred by employers [7]. A morbidly obese employee currently costs employers, on average, an additional US $4000 or more per year than an employee who is of a healthy weight [8]. When achieving 10% weight loss, it increases the likelihood of lowering cholesterol, blood pressure, and risk for diabetes, and even a modest 5% weight reduction can lead to clinically significant decreases in comorbidities associated with overweight and obesity [9-12]. Owing to the extreme impact overweight and obesity have on morbidity, mortality, and the financial state of health care in the United States, the development of effective weight-loss programs is imperative [13-18].

Many employers have taken experts’ recommendations and implemented an employee wellness program; however, these programs are often underused with short-term benefits [19]. Lack of education, personalization, and slow weight loss in Web-based interventions are directly connected to weak adherence and high attrition leading to unsuccessful outcomes [20]. Therefore, initiating programs that are accessible, personalized, easy to use, and interesting to employees is a key factor to achieving successful outcomes that decrease employer health care costs [21].

The Look AHEAD (Action for Health in Diabetes) trial resulted in increased outcomes and greater retention in participants receiving education with an intensive behavior modification plan including nutrition and physical activity over education alone [22-24]. Remote programs are also desired and improve adherence in intensive programs [25]. In recent years, Internet accessibility and technology advancements have positively impacted weight-loss programs through the development of mobile phone apps, Web-based weight-loss methods with both personalized and nonpersonalized approaches, and point-click nutrition and fitness information [26,27]. Successful Web-based programs include a structured approach with a hypocaloric nutrition plan, cognitive behavioral strategies, self-monitoring, and individualized feedback and support [28]. Behavioral weight control approaches that include a comprehensive lifestyle modification program using Wi-Fi scales, mobile phones, or tablets for self-monitoring are shown to be effective in achieving a 7%-10% weight reduction [12,25,29].

Combining in-person support with remote technologies has been shown to significantly increase 5% weight-loss outcomes over remote technologies alone [15]. Remote technology such as mobile phone and tablet apps, wireless activity trackers, and wireless scales allows for convenient self-monitoring of weight, food choices, and activity; however, individualization of a participant’s program and personalized feedback create greater adherence, and adherence is associated with greater retention rates [20,30-33].

Efficacy of structured research projects with commercial and proprietary weight-loss programs lack real-world outcomes, meaning that current populations are being selected by the study staff [6]. This lack of evidence is visible in a systematic review regarding efficacy of commercial and proprietary weight-loss programs released by Gudzune et al [6].

Retrofit is a personalized weight management and disease-prevention solution (see Multimedia Appendix 1). The purpose of this study was to report Retrofit’s weight-loss outcomes at 6, 12, and 24 months using real-world data. A secondary purpose of the study was to characterize behaviors, age, and sex of participants who achieved a weight loss of 10% or greater at 12 months, who are labeled as high performers.

## Methods

### Research Design

A retrospective analysis using deidentified data of the Retrofit weight-loss program was performed using a case series [34] approach that included the participants with known weight measurements at 6, 12, and 24 months. This study characterized the changes in participants’ body weight and body mass index (BMI) from the first weight measurement (start date) to different time points and the percentage of participants who reached a clinically significant weight loss of 5% at the corresponding time period. A secondary analysis was conducted focusing on participants with known weight at 12 months, to characterize the differences in various behaviors, age, and sex between participants who lost ≥10% of their starting weight and remaining participants. Western Institutional Review Board granted institutional review board exemption.

### Subjects

Clients in this study were paying customers of the Retrofit program who enrolled through the direct-to-consumer website (Retrofitme.com) or through an employer-sponsored program. Participants were defined as a client who provided at least one weight measurement (N=2720).

**Figure 1:**
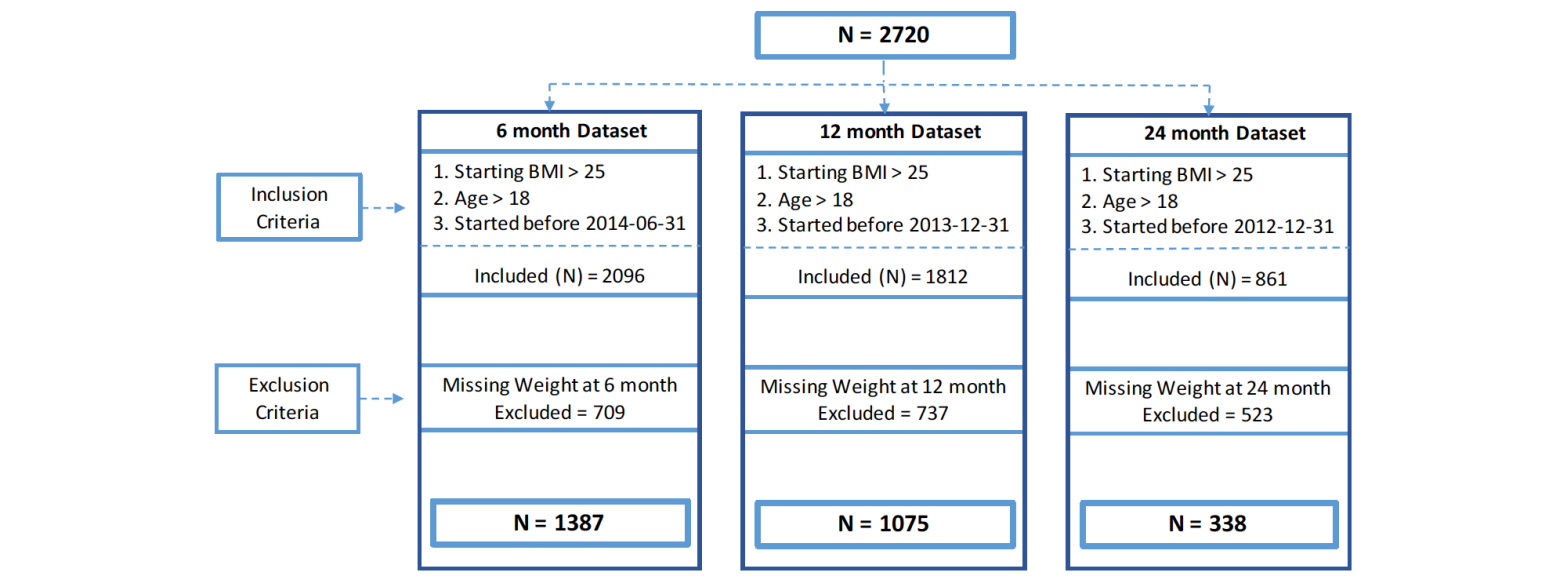
Study population with exclusion, with restrictions at each time point. BMI: body mass index.

Inclusion criteria included participants who had a starting BMI of >25 kg/m², had signed up for the program between September 27, 2011, and December 31, 2014, and were at least 18 years of age. Exclusion criteria included a participant having no weight measurement available at 6, 12, or 24 months. A lack of available weight measurement was due to either a start date more recent than the reviewed data window (see Figure 1, inclusion criteria) or not providing a known weight measurement (see Figure 1, exclusion criteria). Decreasing numbers of participants at each data window was related to study design and not directly related to dropping out of the Retrofit program. The reported Retrofit programs are 12-month programs. However, participants could request to continue their program beyond 12 months. If a participant does not remain in an active program guided by an expert coach, the participant still has continued access to program devices, Wi-Fi scale and activity tracker, and private dashboard. According to previous study observations, self-weighing adherence decreases over time [35-39]; therefore, including only those participants with weight data in this study design, the number of excluded participants increased as the data windows progressed. For the purposes of this study, excluded participants will be defined as dropouts.

Initially, 2720 clients were considered as study participants who provided at least one weight measurement. On the basis of the inclusion and exclusion criteria defined in Figure 1, the final study sample sizes were determined at different time points. At 6 months, 1387 participants met the final inclusion criteria. At 12 months, 1075 participants met the final inclusion criteria. At 24 months, 338 participants met the final inclusion criteria. The sample was treated as an independent group at each data window, as a participant could have a known weight measurement at any one or more than one of the observed milestones. See Figure 1.

### Program Description

Retrofit offered 3 programs during the period of analysis: Expert 10 Weight Loss Program, Expert 15 Weight Loss Program, and Advisor Weight Loss Program. The programs were designed with a 6-month weight-loss phase and an additional 6-month weight maintenance phase. Weight maintenance after the 12 months was anticipated to be observed through continued application of the learned health behaviors.

The participant was initiated into the program, meeting with a personal program advisor who explained the components of the program, and tested familiarity with the provided technology and Web-based video communication capabilities. Client information was collected during the initial setup period.

The participant was provided with a Fitbit activity tracker, Wi-Fi–enabled scale, and access to a private dashboard (see Multimedia Appendix 2). The dashboard allowed each participant to keep a personal food and exercise log, review his or her personal data, and enabled communication between the participant and his or her expert coach through a Web-based electronic messaging feature. The private dashboard was accessed via the Retrofitme.com Web application, mobile website, or mobile phone app, which was available on Apple iOS and Android platforms.

Participants were provided with sessions and check-ins to use with an expert coach during their program. The initial session was scheduled for 60 minutes with follow-up sessions scheduled for 30 minutes. Scheduled check-ins were 15 minutes. Sessions were conducted via Web-based video chat or mobile phone (see Multimedia Appendix 3). All sessions included an educational component, allowing the participant to learn the Retrofit philosophy and weight-loss guiding principles associated with nutrition, mindset, exercise, and daily activity. Sessions and check-ins were used for collaboration between the expert coach and participant to evaluate current health-related behaviors, goal setting, and create individualized plans and strategies. The sessions and check-ins also provided accountability to previously agreed upon strategies. A minimum of 24 one-on-one coaching sessions that included sessions and check-ins, or only check-ins, were allotted to each participant’s program. One-on-one coaching session totals included 24 sessions for Expert 10, 36 sessions for Expert 15, and 12 sessions and 12 check-ins for Advisor.

Participants were encouraged to weigh in and wear their activity tracker daily. Wearing an activity tracker and setting a step goal has been associated with a decrease in BMI and increase in activity [40]. Step count goals were personalized to the participant’s baseline step count. Expert coaches recommended that participants increase step counts in increments of 500 to ultimately achieve their personal daily step goal at 6 months. Participants were encouraged to communicate daily with the expert coach via Web-based messages on the dashboard. Expert coaches were required to review a client’s food and exercise logs, step data, weight data, and progress toward goals a minimum of 1 time per week to provide feedback via Web-based messages. If a client initiated a coaching conversation, the expert coach was required to respond within 24 hours.

All 3 programs provided the participants with the same technology, access to a weight-loss expert, accountability, feedback, and the opportunity to communicate with a weight-loss expert via Web-based messages equally. The differences among programs were defined by number and type of one-on-one coaching sessions a participant was provided, and the Advisor program provided access to 1 expert coach, whereas Expert 10 and Expert 15 programs provided a team of experts in mindset, nutrition, and exercise. Weight-loss experts were employed professionals with a master’s or doctorate level college education in nutritional sciences, exercise physiology, health education, counseling, or psychology. Mindset experts had degrees in counseling, health education, social work, or psychology. Nutrition experts were registered and licensed dietitians, and exercise experts were exercise physiologists. Experts are assigned to a participant for the duration of their program; however, a new expert may have been assigned to a participant’s program related to employee turnover or as part of the quality assurance process. Expert coaches were trained on and utilized Retrofit’s weight-loss protocol.

### Data Collection

Weight data were collected through use of the provided Wi-Fi connected scale (97.87% of recorded weights) or self-reported entry (2.13%). Self-reported entry was permissible if participants had difficulty setting up their Wi-Fi scale. Starting weight was determined at the first collected weight from the participant. The criteria for determining if a participant had a known weight measurement at different time points in the program are outlined in Table 1. A widening window approach was used at each time point to account for decreased self-weighing behavior. A reduction in the frequency of self-weighing behavior is observed in existing research [35-39]. Therefore, a wider range of time was used to determine a participant’s weight at 12 and 24 months. Similar widening window approaches have been used in previous commercial weight-loss studies [41].

When more than one measurement was collected within the identified range, the weight measurement on or closest to the specified day of measurement was used. Participants were encouraged to step on the scale daily.

**Table 1:** Criteria for identifying participants’ weight at specific time points in the program.

Accepted days for selecting known weight	Milestone		
	6 Months	12 Months	24 Months
Target	Day 180	Day 360	Day 720
Range	Days 159-187	Days 300-367	Days 660-727

Participant self-monitoring adherence was analyzed at 12 months through the use of the activity tracker and frequency of weigh-ins. Participant behaviors and engagement were observed through the number of coaching conversations in the form of an electronic message posted on the private dashboard between participant and weight-loss expert, number of days that an electronic message was logged, along with the length of each electronic message. Coaching conversations include both coach-initiated and participant-initiated Web-based messages. In addition, number of coaching sessions attended were also analyzed for participant engagement.

### Analysis

The primary outcome measurements were total weight lost in kilograms, percentage of weight lost, change in BMI, and percentage of participants who lost ≥5% and ≥10% of their starting weight. Program outcomes were analyzed at 6, 12, and 24 months, grouped by sex. At each milestone, data of participants with a known weight measurement were used to calculate outcomes.

Further analysis summarized self-monitoring behaviors and coaching conversations via Web-based messages between the participant and weight-loss expert at 12 months. The analysis grouped participants based on sex. Participants were also divided into groups that lost at least 10% of their starting weight at 12 months and those who had a weight loss of less than 10% at 12 months.

The summarized behaviors include total weigh-in measurements, total days of activity tracker use, daily step count average, total number of coaching conversations via Web-based messages, total count of days with a conversation, average conversation length, and number of coaching sessions attended.

**Figure 2:**
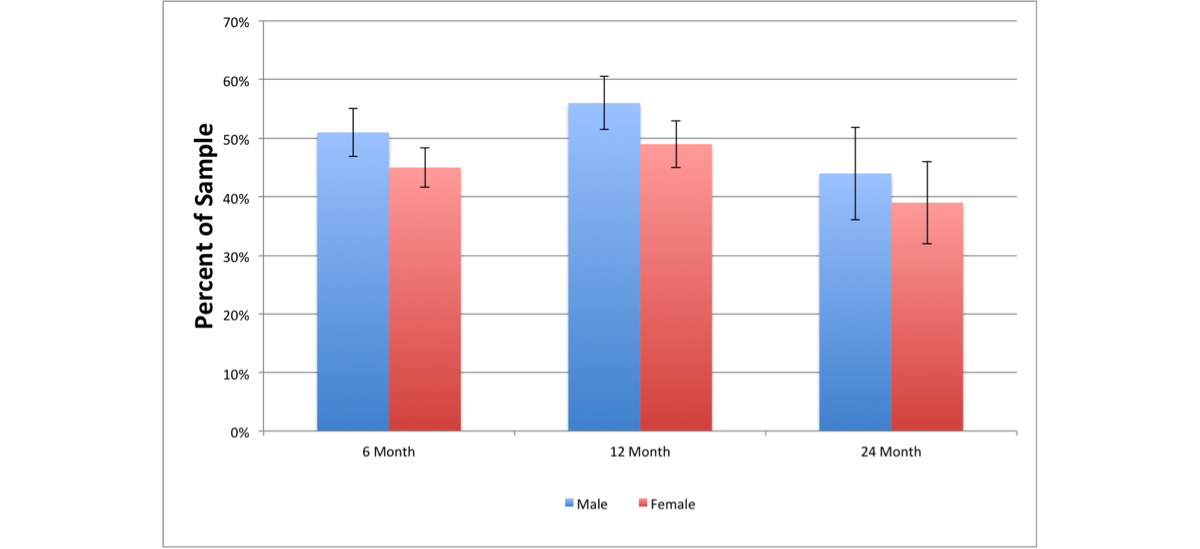
Percentage of male and female participants who lost ≥5% of starting weight at 6, 12, and 24 months. Error bars indicate 95% confidence intervals.

Primary data analyses were performed using Python 2.7.11, which included NumPy 1.10.4, Pandas 0.17.1, and SciPy 0.17.0 analytic packages. For two-group comparisons, *t* tests of equal variance were conducted on continuous variables at baseline and subsequent time points. One-way analysis of variance (ANOVA) was used to determine mean differences for more than two-group comparisons. Subsequently, Tukey tests were conducted to determine mean differences. Chi-square analyses were performed to determine differences among categorical variables when appropriate. Outcome variable means are summarized with standard errors (SEs) and a 95% confidence interval is included in the populations summarized in Figure 2. Alpha was set at .05 for all statistical tests to determine statistical significance.

## Results

### Baseline Characteristics

Of the participants with a known weight measurement at each time point, 41.9% (581/1387) at 6 months, 43.6% (469/1075) at 12 months, and 44.4% (150/338) at 24 months were male. There were no differences in age or starting BMI at baseline, between male and female participants, for each sample. Male participants had a higher starting weight at 6 months (*P*<.001), 12 months (*P*<.001), and 24 months (*P*<.001). Baseline summaries for age, starting weight, and starting BMI are outlined in Table 2.

**Table 2:** Baseline demographics.

Demographics	6 Months, mean (SD)		*P* value	12 Months, mean (SD)		*P* value	24 Months, mean (SD)		*P* value
	Male (n=581)	Female (n=806)		Male (n=469)	Female (n=606)		Male (n=150)	Female (n=188)	
Age, years	47.3 (11.3)	47.2 (10.8)	.85	47.6 (11.4)	47.6 (10.8)	.99	48.3 (12.0)	48.3 (11.2)	.99
Starting weight, kilograms	109.5 (22.2)	91.3 (20.2)	<.001	108.9 (22.0)	90.9 (19.5)	<.001	105.3 (19.6)	88.47 (17.1)	<.001
Starting BMI^a^, kg/m^2^	33.8 (6.4)	33.5 (7.1)	.40	33.6 (6.4)	33.4 (6.8)	.61	33.0 (6.1)	32.9 (6.0)	.87

^a^BMI: body mass index.

### Weight Change Status

The average weight loss at 6 months was −5.55% (SE 0.20) and −4.86% (SE 0.18) for males and females, respectively. Males at 12 months had an average weight loss of −6.28% (SE 0.28). Females at 12 months lost an average of −5.37% (SE 0.28). The average weight loss at 24 months was −5.03% (SE 0.61) and −3.15% (SE 0.62) for males and females, respectively. Weight loss was calculated by subtracting baseline weight from milestone weight. There was a significant difference in total weight lost in kilograms, percentage of weight lost, and BMI change at all of the observed milestones, when comparing males and females. A complete outline of these weight change outcomes, for all 3 observed durations is available in Table 3.

Figure 2 shows the percentage of the male and female participants who lost ≥5% of their starting weight. There was a significant difference between male and female participants at 6 months (*P*=.045) and 12 months (*P*=.02).

### Baseline Characteristics for High-Performing and Remaining Participants

Participants who lost ≥10% of their starting weight were identified as high performers. Those participants who did not achieve that amount of weight loss were identified as the remaining participants.

At baseline, high-performing males had a statistically significant higher average starting weight of 112.7 (SD 22.11) kg, whereas the remaining male participants had an average starting weight of 107.8 (SD 21.84) kg (*P*=.045). High-performing females were older on average than the remaining females, where the average ages were 50.3 (SD 11.0) years and 46.9 (SD 10.7) years, respectively (*P*=.001). All other baseline characteristics were similar between the high-performing participants and the remaining participants. Baseline characteristics are outlined in Table 4.

**Table 3:** Weight-loss outcomes.

Outcomes	6 Months		*P* value	12 Months		*P* value	24 Months		*P* value
	Male (n=581)	Female (n=806)		Male (n=469)	Female (n=606)		Male (n=150)	Female (n=188)	
Weight change, kilograms mean (SE)	-6.17 (0.24)	-4.44 (0.17)	<.001	-7.03 (0.34)	-4.90 (0.26)	<.001	-5.68 (0.76)	-2.78 (0.58)	0.002
Weight change, % mean (SE)	−5.55 (0.20)	−4.86 (0.18)	.01	−6.28 (0.28)	−5.37 (0.28)	.02	−5.03 (0.61)	−3.15 (0.62)	.03
BMI^a^ change, kg/m^2^ mean (SE)	−2.09 (0.08)	−1.83 (0.06)	.009	−2.37 (0.11)	−1.99 (0.10)	.009	−2.04 (0.24)	−1.28 (0.22)	.02
% With ≥5% weight loss % (n/N)(SE)	50.6% (294/581) (2.07)	45.2% (364/806) (1.75)	.045	56.3% (264/469) (2.29)	48.8% (296/606) (2.03)	.02	44.0% (66/150) (4.05)	39.4% (74/188) (3.55)	.39
% With ≥10% weight loss % (n/N)(SE)	17.2% (100/581) (1.23)	14.3% (115/806) (1.57)	.16	22.4% (105/469) (1.92)	21.9% (133/606) (1.68)	.92	16.7% (25/150) (3.04)	20.7% (39/188) (2.96)	.42

^a^BMI: body mass index.

**Table 4:** Characteristics of high performers versus remaining participants at 12 months.

Characteristics	Male, mean (SD)		*P* value	Female, mean (SD)		*P* value
	High performers (n=105)	Remaining (n=364)		High performers (n=133)	Remaining (n=473)	
Age, years	46.9 (11.3)	47.9 (11.4)	.44	50.3 (11.0)	46.9 (10.7)	.001
Starting weight, kilograms	112.7 (22.11)	107.8 (21.84)	.045	91.1 (18.2)	90.8 (19.9)	0.87
Starting BMI^a^, kg/m^2^	34.6 (6.37)	33.3 (6.37)	.06	33.3 (5.9)	33.4 (7.05)	.94

^a^BMI: body mass index.

**Figure 3:**
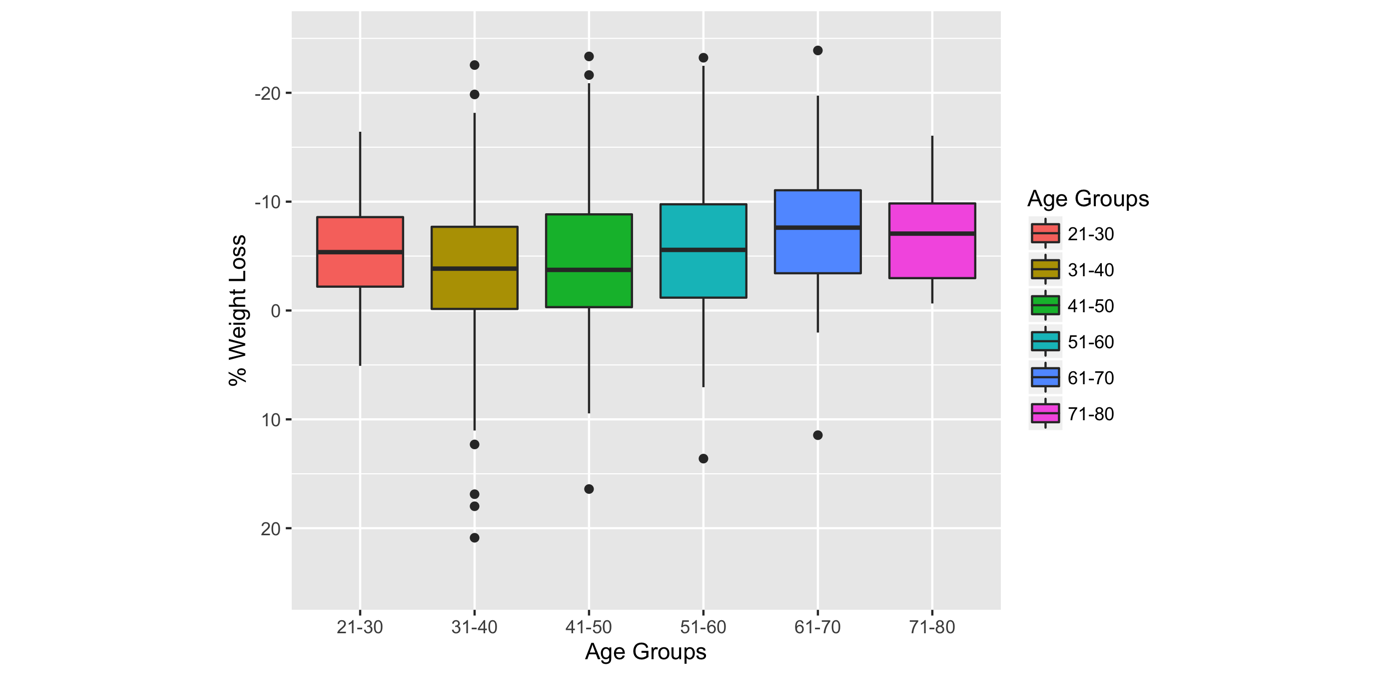
One-way analysis of variance: female age groups and percentage of weight lost (*P*=.002). The bold horizontal line is the median, the bottom and top borders of the boxes are 25th and 75th percentiles, respectively; the vertical lines below and above the boxes extend up to 2.5th and 97.5th percentiles, respectively; the black circles are outliers.

Further analysis of participants’ sex, age, and average weight loss at 12 months was performed by dividing participants by sex, grouping them by 10-year age ranges, and conducting a one-way ANOVA. To address outlying age groupings, participants 20 years and younger or 80 years and older were not included in the assessment. For male participants, there was no statistically significant difference (*P*=.37) in relation to age and weight loss. However, for female participants, there was a significant difference of mean weight loss between the different groups (*P*=.002), see Figure 3. A subsequent Tukey test was performed, finding that the significant mean differences occurred between the 31- to 40-year age group and 51- to 60-year age group (*P*=.026) and between the 31- to 40-year age group and 61- to 70-year age group (*P*=.004).

### Self-Monitoring Behaviors and Coaching Conversations at 12 Months

For all self-monitoring behaviors, the high-performing participants had significantly higher adherence at 12 months. High-performing males weighed in 197.3 (SE 9.97) times, whereas remaining males weighed in 165.4 (SE 4.39) times (*P*=.001). High-performing females weighed in 222 (SE 8.47) times compared with 167 (SE 4.12) times for remaining females (*P*<.001). The total days of activity tracker use and average steps per day for high-performing females was 304.7 (SE 6.81) days and 8380.9 (SE 268.5) steps, respectively, whereas the remaining females had 266.6 (SE 98.1) days (*P*<.001) and 7059.7 (SE 2499.9) steps (*P*<.001). Males had 297.1 (SE 88.0) and 255.3 (SE 106.3) activity tracker days (*P*<.001) for high performers and remaining participants, respectively. On average, high-performing males had 9099.3 (SE 2954) average daily step counts, whereas the remaining males had 8251.4 (SE 2821) steps (*P*=.008). Self-monitoring measurements for males and females are outlined in Table 5.

**Table 5:** Self-monitoring and engagement.

	Male, mean (SE)		*P* value	Female, mean (SE)		*P* value
	High performers (n=105)	Remaining (n=364)		High performers (n=133)	Remaining (n=473)	
Weigh-in days^a^	197.3 (9.97)	165.4 (4.39)	.001	222 (8.47)	167 (4.12)	<.001
Activity tracker days^b^	297.1 (8.71)	255.3 (5.60)	<.001	304.7 (6.81)	266.6 (4.52)	<.001
Daily step count	9099.3 (289.7)	8251.4 (148.7)	.008	8380.9 (268.5)	7059.7 (115.1)	<.001
Total coaching conversations^c^	260.2 (14.02)	236 (7.87)	.14	341.4 (15.7)	301.1 (8.9)	.03
Coaching conversation days^d^	100.9 (3.60)	95.9 (2.21)	.27	118 (4.10)	108 (2.17)	.03
Coaching conversation length^e^	239.1 (6.93)	262 (5.13)	.03	232.4 (5.31)	247.6 (3.61)	.04
Number of coaching sessions attended^f^	18.7 (0.62)	16.7 (0.43)	.008	19.7 (0.57)	17.7 (0.34)	.003

^a^Weigh-in day: day where participant reported weigh-in via Wi-Fi scale or self-report.

^b^Activity tracker day: day where participant’s activity tracker recorded more than one step count.

^c^Coaching conversation: online communication between expert and participant in the form of an electronic message, excludes communication between coach and participant in live one-on-one coaching sessions.

^d^Coaching conversation days: a day where a participant or expert posted an electronic message.

^e^Coaching conversation length: the number of characters of an electronic message.

^f^Number of coaching sessions attended: the total number of one-on-one coaching sessions a participant attended, includes both 30-minute sessions and 15-minute check-ins.

Reviewing engagement between the participant and expert coach, high-performing female participants had significantly more total coaching conversations via Web-based messages with 341.4 (SE 15.7) compared with 301.1 (SE 8.88) for the remaining participants (*P*=.03). High-performing females also had more days with at least one coaching conversation, with 118 (SE 4.10) days compared with the remaining participants who had 108 (SE 2.17) days (*P*=.03). Similar trends were found when reviewing male participant coaching conversation totals; however, these were not significant. High performers had 260.2 (SE 14.02) coaching conversations and 100.9 (SE 3.60) days, compared with the remaining males with 236 (SE 7.87) coaching conversations (*P*=.14) and 95.9 (SE 2.21) days (*P*=.27). Interestingly, the coaching conversations were not longer for high performers. This was true for females where conversation length was 232.4 (SE 5.31) characters for high performers and 247.6 (SE 3.61) characters for remaining participants (*P*=.04). This also was found with males, as high performers’ conversation length was 239.9 (SE 6.93) characters compared with 262 (SE 5.13) characters (*P*=.03) for remaining participants. Coaching conversations via electronic message measurements can be found in Table 5.

Further engagement was reviewed in number of coaching sessions attended. High-performing male participants attended 18.7 (SE 0.62) sessions compared with remaining male participants attending 16.7 (SE 0.43) sessions (*P*=.008). High-performing female participants attended 19.7 (SE 0.57) sessions, whereas remaining female participants attended 17.7 (SE 0.34) sessions (*P*=.003). High-performing male and female participants attended a statistically significant number of coaching sessions over remaining participants. See Table 5 for number of coaching session measurements.

## Discussion

### Principal Findings

Participants on the Retrofit program lost an average of −5.21% (male −5.55%, female −4.86%) at 6 months, −5.83% (male −6.28%, female −5.37%) at 12 months, and −4.09% (male −5.03%, female −3.15%) at 24 months. Men consistently lost more weight than women at all the milestones. At 12 months, 56.3% (264/469) of males and 48.8% (296/606) of females had clinically significant weight loss, losing 5% of starting weight. High-performing male and female participants, who lost ≥10% of their starting weight, had higher adherence to all self-monitoring behaviors, whereas only high-performing female participants had a higher rate of engagement through coaching conversations. However, both male and female high performers attended a statistically significant number of one-on-one coaching sessions.

### Male Versus Female Outcomes

Although more females were included in the study population at each time point, no differences were observed in age or starting BMI at baseline; however, males had a higher starting weight at each time point. In addition, men were significantly more successful at 6, 12, and 24 months with more total weight lost in kg, greater percentage of weight loss, and change in BMI. Therefore, more male participants lost ≥5% than female participants. Enrollment, baseline data, and weight loss comparison between male and female participants are consistent with other weight-loss studies; however, a higher percentage of men were represented in the Retrofit study population than presented in the literature [6,9,15,17,19,20,22,23,30,33,40,42]. Women are shown to seek out weight-loss opportunities more than men, whereas men lose more weight regardless of age or baseline weight characteristics, likely due to biological differences between males and females [5,42,43]. However, regardless of total weight lost, losing a clinically significant amount of weight at ≥5% is of most importance to reduce comorbidities related to overweight and obesity [9-12,25,42,43].

### High Performers’ Characteristics and Behaviors

Determining potential baseline indicators and behaviors of participants achieving 10% or greater weight loss was important to increase the reduction of overweight- and obesity-related health conditions [9-11,23]. A majority of the baseline characteristics for high-performing males and females were not significantly different compared with the remaining participants. However, high-performing males had a higher starting weight than remaining male participants and high-performing females were older on average than the remaining female participants. Older adults have been shown to be more successful in losing weight than younger adults owing to intervention adherence in the Look AHEAD trial [42,43]. Men, in general, and specifically older women are more motivated by health risks than cosmetic or social factors [44,45].

Of particular interest were the participant behaviors associated with high-performing participants at 12 months, which included the self-monitoring behaviors of weighing in, number of days wearing an activity tracker, and average number of steps per day, as well as engagement behaviors, including total number of coaching conversations, number of days with a coaching conversation, length of coaching conversations, and number of coaching sessions attended. Male and female high performers had a greater adherence rate to all self-monitoring behaviors and attended significantly more coaching sessions, which was consistent with the large amount of available research connecting program adherence to weight loss [20,25,33,44]. Self-monitoring behaviors, specifically when incorporated through technology, have consistently been shown to improve weight-loss outcomes [20,25,28,30,32,33].

High-performing female clients engaged more through coaching conversations than high-performing male clients on both total number of conversations and number of days with a conversation. This also was seen by Tate et al [33] in number of diary submissions being significantly associated with weight loss, although the study did not divide participants by sex. Participants, both male and female, who did not achieve 10% weight loss had longer conversations than high-performing participants. This observation identifies the hypothesis that frequency of messages, as opposed to length of messages, is a critical component of participant and coach asynchronous communication via Web-based messages. Multiple studies support the hypothesis that frequency of contact does improve weight-loss outcomes, specifically in achieving ≥5% weight loss [15,31,32].

### Strengths and Limitations

This study has several strengths, including reporting of real-world weight-loss outcomes. Participants were actual clients of Retrofit and were not recruited or provided with any incentives to participate in the study. In addition, all clients who met the starting BMI, age, and weight inclusion criteria were included as participants. No client who had a lack of success on the program was removed or eliminated from the population. As an uncommon research practice noted by Gudzune et al [6], researchers conclude that this study adds value and brings a unique set of outcomes to weight-loss research. No previous commercial program has published all of its data in such a manner, providing a true picture of efficacy of the Retrofit program. In addition, outcomes were segmented by sex to identify specific baseline characteristics and behaviors for success between men and women; and lastly, age was used as an additional component to target baseline characteristics and behaviors to achieve 10% or greater weight loss in 12 months.

In addition to the identified strengths, the researchers also noted some weaknesses. According to the study design, cross-sectional samplings were performed at 6, 12, and 24 months to select participants with known weight, which provided a separate population for each weight-loss period. The case series approach of this study does not allow any causal inferences based on the critical observations. In addition, because of the real-world population, it is unknown if participants were integrating any other weight-loss practices outside of the Retrofit program components.

### Future Research

Because of a lack of real-world outcomes within the commercial weight-loss industry, Retrofit encourages all commercial weight-loss programs to publish similar data to show efficacy of programs. By reporting real-world outcomes in relation to targeted behaviors, commercial weight-loss programs can structure protocols and client strategies to enhance long-term weight-loss success. Clearly defining the necessary behaviors for long-term weight-loss success and the efficacy of each commercial weight-loss program will solidify our ability as an industry and country to combat the obesity crisis, including obesity-related diseases such as heart disease and diabetes.

Recommended future research includes studying a population over time for a causal effect of weight loss, as well as comparing the population with a control group and targeting specific characteristics and behaviors for high-performing clients such as what engagement factors matter for male participants, why older female participants are more successful, and why men with a higher starting weight are more successful. In addition, it is recommended to compare male and female high performers as a single population with the remaining participants for further insight into baseline characteristics and behaviors for success, and observing self-monitoring and health behavior adherence beyond 12 months. Finally, evaluate the impact of Retrofit’s weight-loss program on short-term and long-term employer health care spending.

### Conclusions

In conclusion, participants on the Retrofit program lost an average of −5.21% at 6 months, −5.83% at 12 months, and −4.09% at 24 months. Men, on average, lost more weight than women. High-performing participants, or participants who lost ≥10% of starting weight at 12 months, had a greater adherence to the self-monitoring behaviors of weighing in, number of days wearing an activity tracker, and average number of steps per day. Female high performers had a higher engagement in coaching conversations through conversation days and total number of conversations with their expert coaches. However, both high-performing male and female participants attended significantly more one-on-one coaching sessions.

## Appendix

Multimedia Appendix 1 Retrofit logo.

Multimedia Appendix 2 The technology provided included a Wi-Fi–enabled scale, activity tracker, and access to a private dashboard. The dashboard was accessible via Web and mobile apps.

Multimedia Appendix 3 Coaching sessions were conducted via video chat, available online or with a mobile phone.

## Abbreviations

ANOVA: analysis of variance
BMI: body mass index
Look AHEAD: Action for Health in Diabetes
SE: standard error
