# Supplementary figures and images for: Retrofit Weight-Loss Outcomes at 6, 12, and 24 Months and Characteristics of 12-Month High Performers: A Retrospective Analysis
Source: JMIR Mhealth Uhealth. 2016 Aug 22;4(3):e101. doi: 10.2196/mhealth.5873 (PMC5011555; doi:10.2196/mhealth.5873)

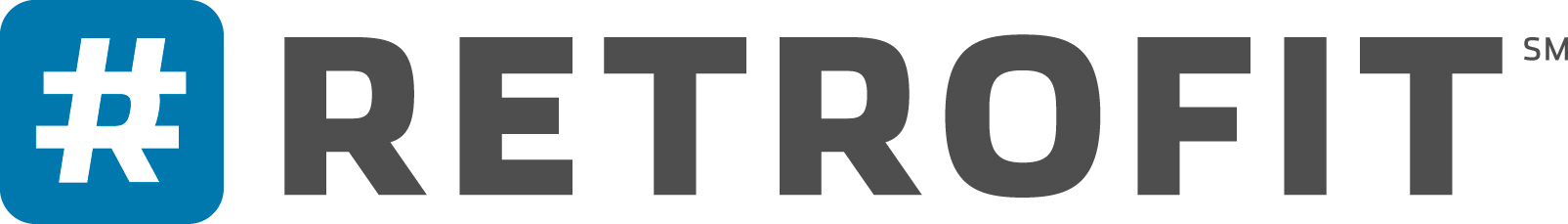

Supplement: Multimedia Appendix 1 [file mhealth_v4i3e101_app1.jpg]

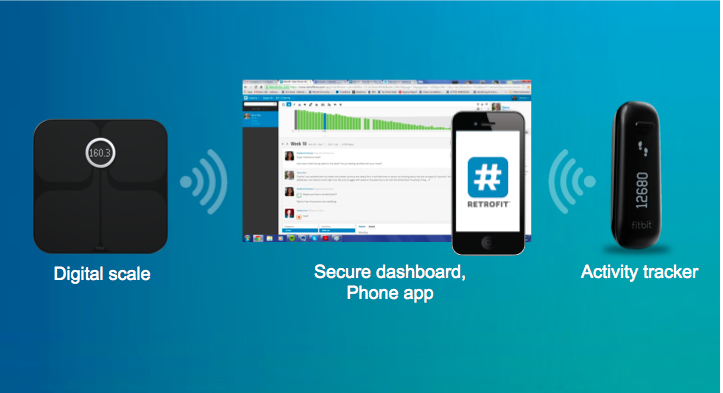

Supplement: Multimedia Appendix 2 [file mhealth_v4i3e101_app2.png]

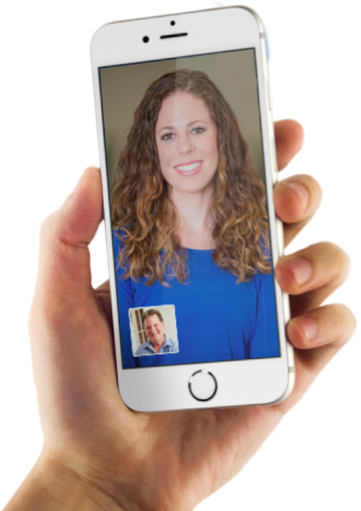

Supplement: Multimedia Appendix 3 [file mhealth_v4i3e101_app3.png]
